# Supplementary material for: Manipulating solvent fluidic dynamics for large-area perovskite film-formation and white light-emitting diodes
Source: Nat Commun. 2024 Feb 5;15:1066. doi: 10.1038/s41467-024-45488-5 (PMC10844237; doi:10.1038/s41467-024-45488-5)
Supplement: Supplementary file 1 — Supplementary Information [file 41467_2024_45488_MOESM1_ESM.pdf]

Supplementary information for

Manipulating solvent fluidic dynamics for large-area perovskite film-formation and white light-emitting diodes

Guangyi Shi[1†], Zongming Huang[1†], Ran Qiao[2†], Wenjing Chen[1], Zhijian Li[1], Yaping Li[3], Kai Mu[2], Ting Si[2], Zhengguo Xiao[1*]

[1]Department of Physics, CAS Key Laboratory of Strongly coupled Quantum Matter Physics, University of Science and Technology of China, Hefei, Anhui 230026, China.

[2]Department of Modern Mechanics, University of Science and Technology of China, Hefei, Anhui 230026, China.

[3]Center for Micro- and Nanoscale Research and Fabrication, University of Science and Technology of China, Hefei, Anhui 230026, China

[†]These authors contributed equally to this work

[*]Correspond to: zhengguo@ustc.edu.cn

Table of Contents

Supplementary Method 1

Supplementary Figs. 1-18

Supplementary Table 1

Supplementary References 1-2

Supplementary Method 1. Simulation of solvent flow

The simulations in this work are carried out using the COMSOL platform based on the finite element method. Several multiphase models have been developed to simulate the fluid dynamics and capture the movement of the interface. Among these models, the phase-field method is widely used because it can easily deal with the moving contact line problem by considering the diffusion of the interface[1,2]. In addition, the spreading and wetting phenomena of droplets on the substrate are axisymmetric in geometry. Therefore, the laminar 2D-axisymmetric two-phase flow model coupled with the phase-field method is utilized in this work.

1. Computation configuration

The computational domain is shown in **Supplementary Fig. 2**. The width of the computation domain ranges from 0 cm to 4 cm, and its height ranges from 0 cm to 1.2 cm. A liquid droplet is located at the corner of the domain, surrounded by the gas environment. The liquid phase is n-octane, and the gas phase is air. The densities and dynamic viscosity of n-octane are 952 kg m^{-2} and 1 kg m^{-2}, and those of the air surroundings are 4×10^{-4} Pa s and 1.8×10^{-5} Pa s, respectively. The surface tension between the liquid and gas is 20 mN m^{-1}. The initial contact angle θ between the liquid surface and the substrate is 20°. Triangle nodes with sizes of approximately 0.02 mm are used to discretize the computation domain.

2. Governing equations

The conservation equations of mass and momentum are used to simulate the fluid dynamics, including

$$\nabla \cdot \mathbf{u} = 0 \tag{1}$$

$$\rho\left(\frac{\partial \mathbf{u}}{\partial t} + \mathbf{u}\cdot\nabla\mathbf{u}\right) = -\nabla p + \nabla\left\{\mu\left[\nabla\mathbf{u} + (\nabla\mathbf{u})^{\mathrm{T}}\right]\right\} + \mathbf{F} \tag{2}$$

where $\boldsymbol{u}$ is the velocity vector, ρ is the density, p is the pressure, μ is the viscosity, and $\boldsymbol{F}$ is the body force. For this two-phase problem, the density and viscosity are calculated by the volume fraction ϕ

$$\rho = \rho_1 V_{\mathrm{f}1} + \rho_2 V_{\mathrm{f}2} \tag{3}$$

$$\mu = \mu_1 V_{\mathrm{f}1} + \mu_2 V_{\mathrm{f}2} \tag{4}$$

$$V_{\mathrm{f}1} = \frac{1-\phi}{2} \tag{5}$$

$$V_{\mathrm{f}2} = \frac{1+\phi}{2} \tag{6}$$

where the subscript i=1,2 stands for the liquid and the gas phase, respectively.

3. Phase-field method

The phase-field method is employed to capture the interface, which is a diffusion layer with finite thickness and obeys thermodynamically consistent conservation laws. The governing equation of interface development is

$$\frac{\partial \phi}{\partial t} + \mathbf{u} \cdot \nabla \phi = \nabla \cdot \frac{\gamma \lambda}{\varepsilon_{\mathrm{pf}}^2} \nabla \psi \tag{7}$$

where γ is the mobile parameter, λ is the mixing energy density, ψ is the phase-field help variable, and ε_{pf} is the parameter of controlling the thickness of the diffuse interface. These parameters are calculated by

$$\gamma = \chi \varepsilon_{\mathrm{pf}}^2 \tag{8}$$

$$\lambda = \frac{3\varepsilon_{\mathrm{pf}} \sigma}{\sqrt{8}} \tag{9}$$

$$\psi = -\nabla \cdot \varepsilon_{\mathrm{pf}}^2 \nabla \phi + \left(\phi^2 - 1\right)\phi + \frac{\frac{\varepsilon_{\mathrm{pf}}}{\lambda} \partial f}{\partial \phi} \tag{10}$$

where χ is the mobility tuning parameter and σ is the surface tension. The effect of surface tension on fluid dynamics is treated as a body force by

$$\mathbf{F} = \left(\frac{\lambda}{\varepsilon_{\mathrm{pf}}^2} - \frac{\partial f}{\partial \phi} \right) \nabla \phi \tag{11}$$

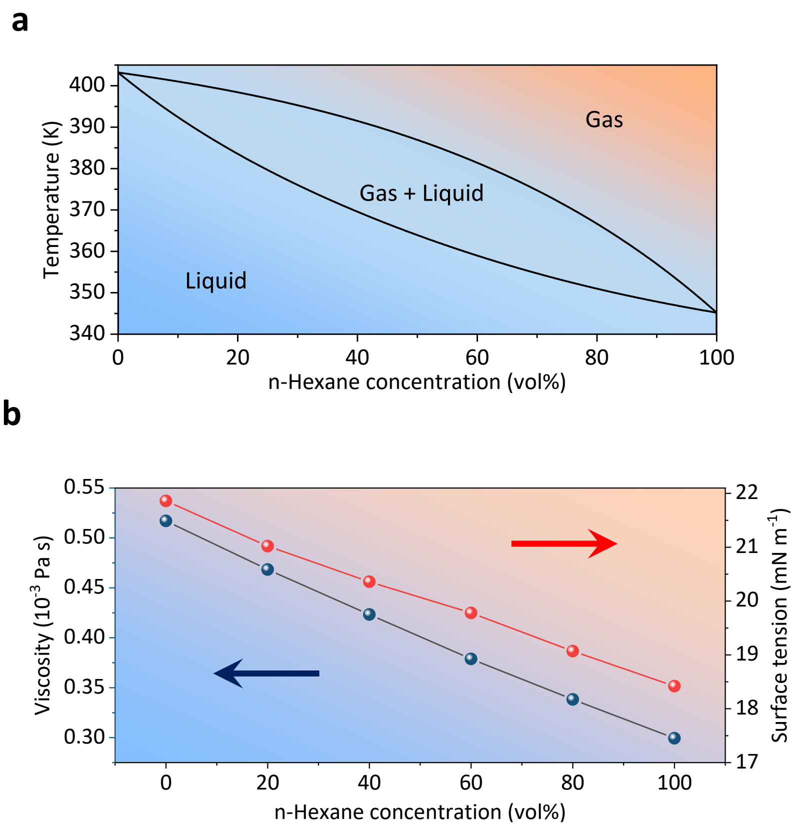

Supplementary Fig. 1 | Physical character of perovskite quantum dot (PQD) inks. a,b, Vapor-liquid equilibrium phase diagram (**a**), surface tension and viscosity (**b**) of PQD inks (30 mg ml^{-1}) with various n-hexane concentrations. Vol% is the volume percent.

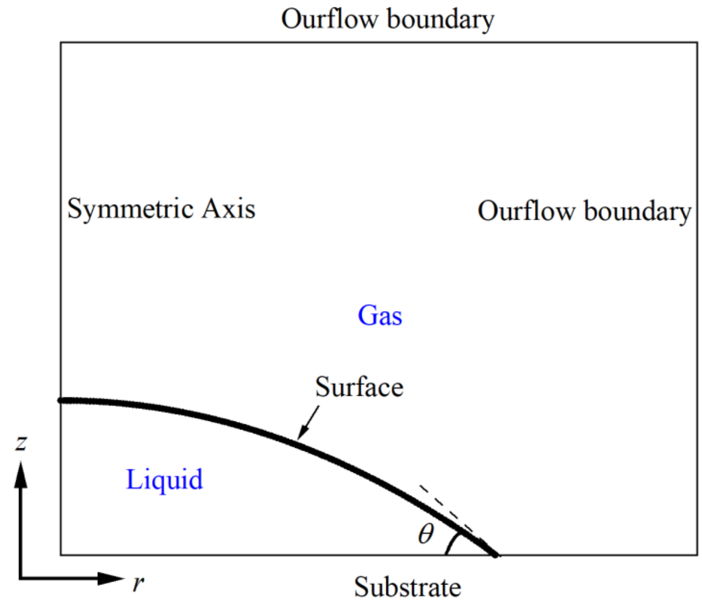

Supplementary Fig. 2 | Computation configuration of the droplets.

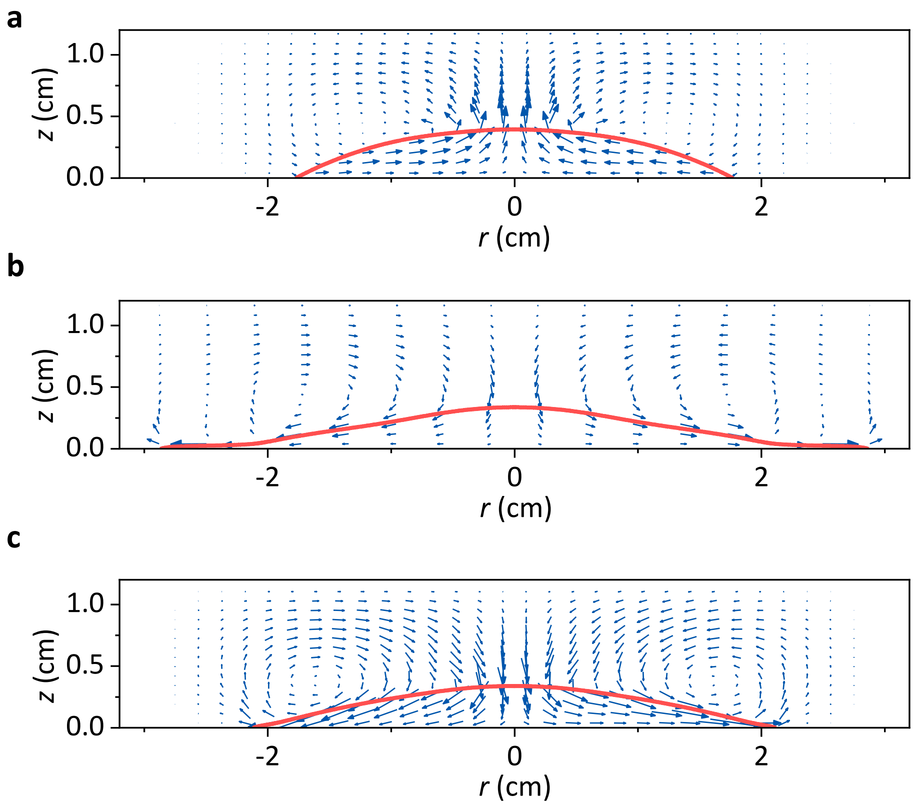

Supplementary Fig. 3 | Simulation of solvent flow. a-c, Simulation (side view) of velocity vector distribution when the perovskite quantum dot (PQD) ink droplets reach equilibrium contact angle with an n-hexane ratio of 0 volume percent (vol%) (**a**), 20 vol% (**b**), and 80 vol% (**c**). The blue arrows represent the velocity vector, and the red circle represents the interface position.

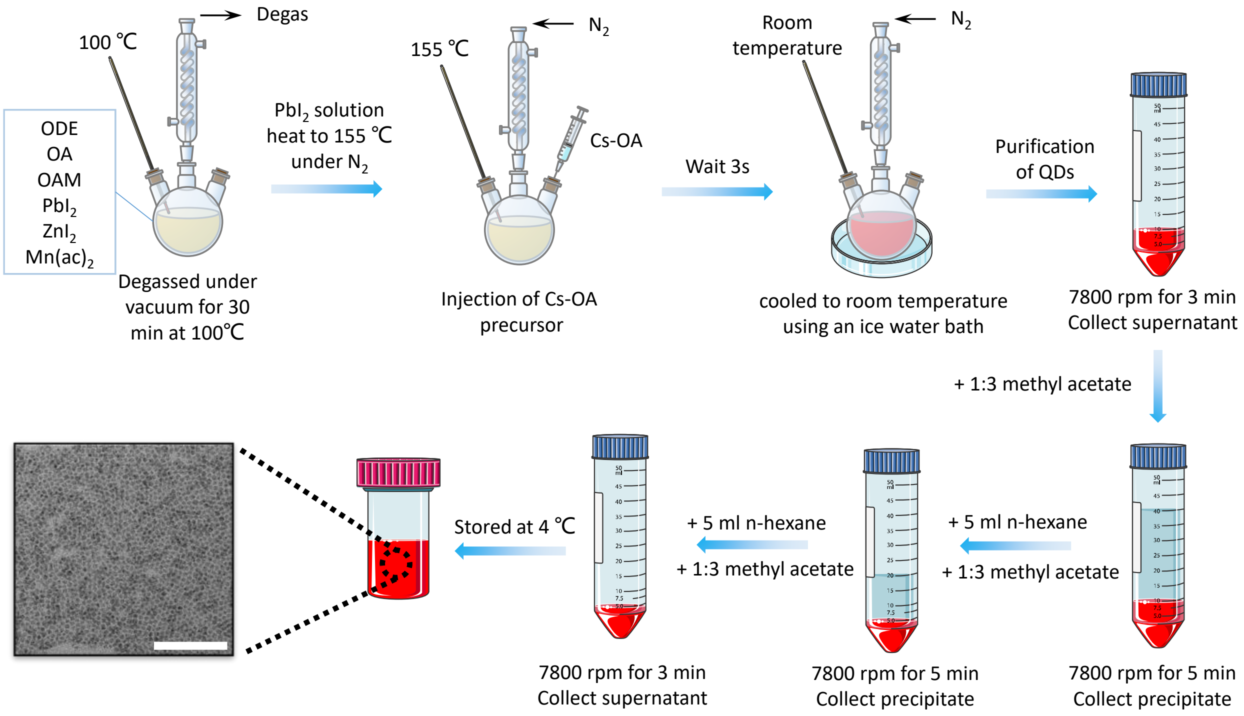

Supplementary Fig. 4 | Synthesis of perovskite quantum dots (PQDs). Schematic of the synthesis and purification of $CsPbI_3$ QDs. The inset shows a TEM image of $CsPbI_3$ QDs and the scale bar is 100 nm.

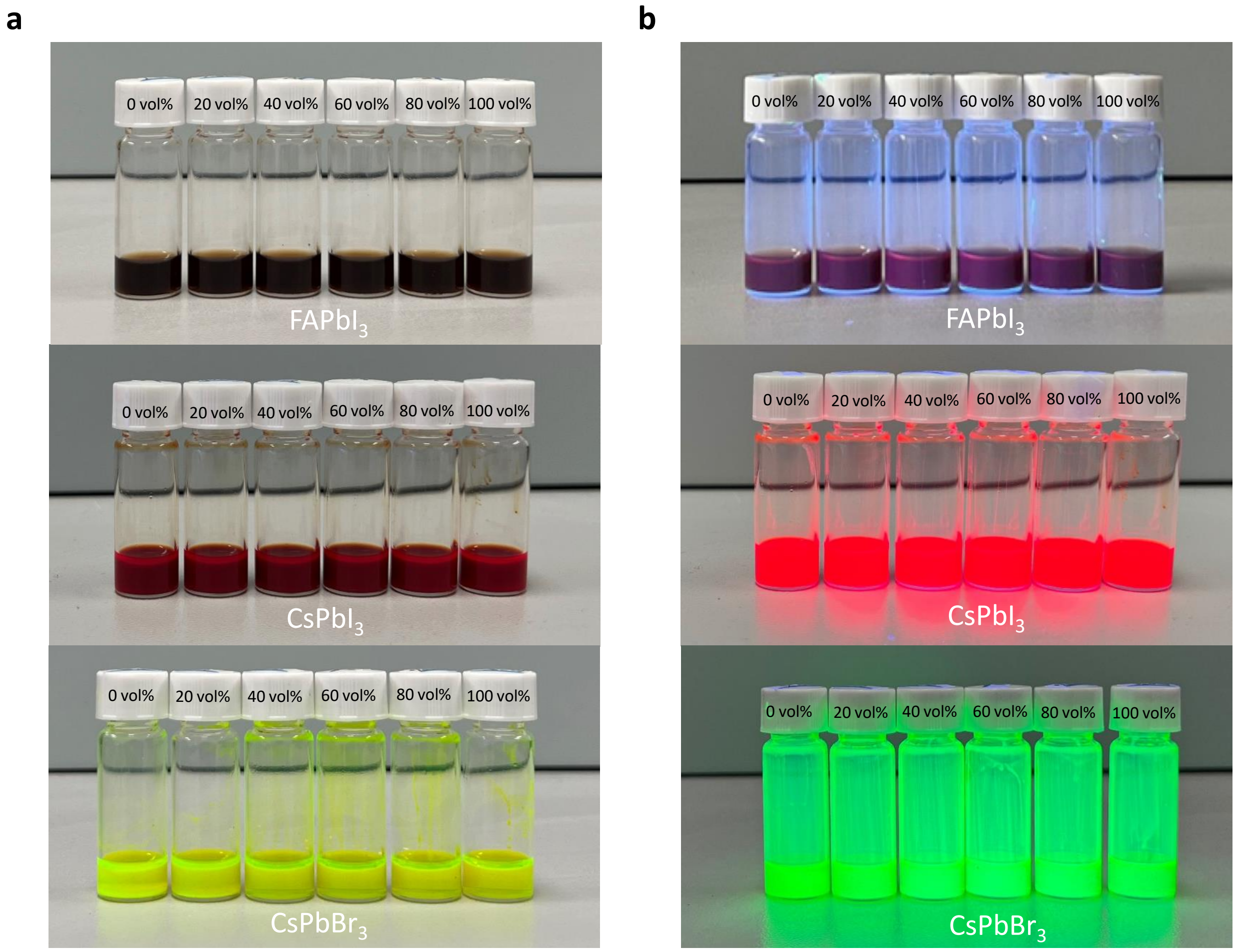

Supplementary Fig. 5 | Photographs of perovskite quantum dot (PQD) inks. a,b, Photographs of $FAPbI_3$, $CsPbI_3$, and $CsPbBr_3$ QDs dispersed in a single solvent system or binary-solvent system with different n-hexane concentrations under room light (**a**) and an ultraviolet (UV) lamp at 365 nm (**b**). Vol% is the volume percent. The concentration of PQDs was 30 mg ml^{-1}.

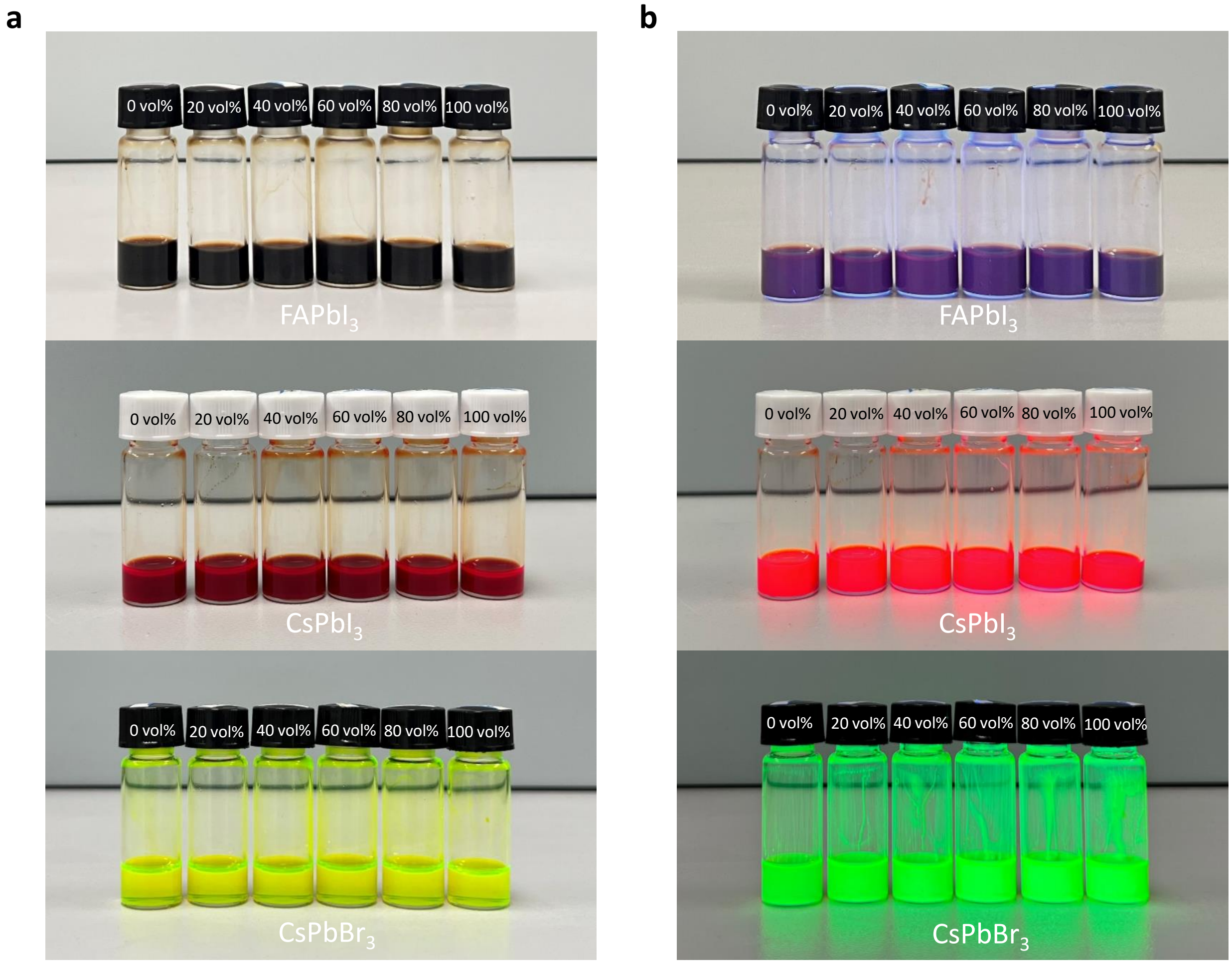

Supplementary Fig. 6 | Aging test of perovskite quantum dot (PQD) inks. a,b, Photographs of $FAPbI_3$, $CsPbI_3$, and $CsPbBr_3$ QDs under room light (**a**) and an ultraviolet (UV) lamp at 365 nm (**b**) after aging in air at 4 °C for 30 days. Vol% is the volume percent. The concentration of PQDs was 30 mg ml^{-1}.

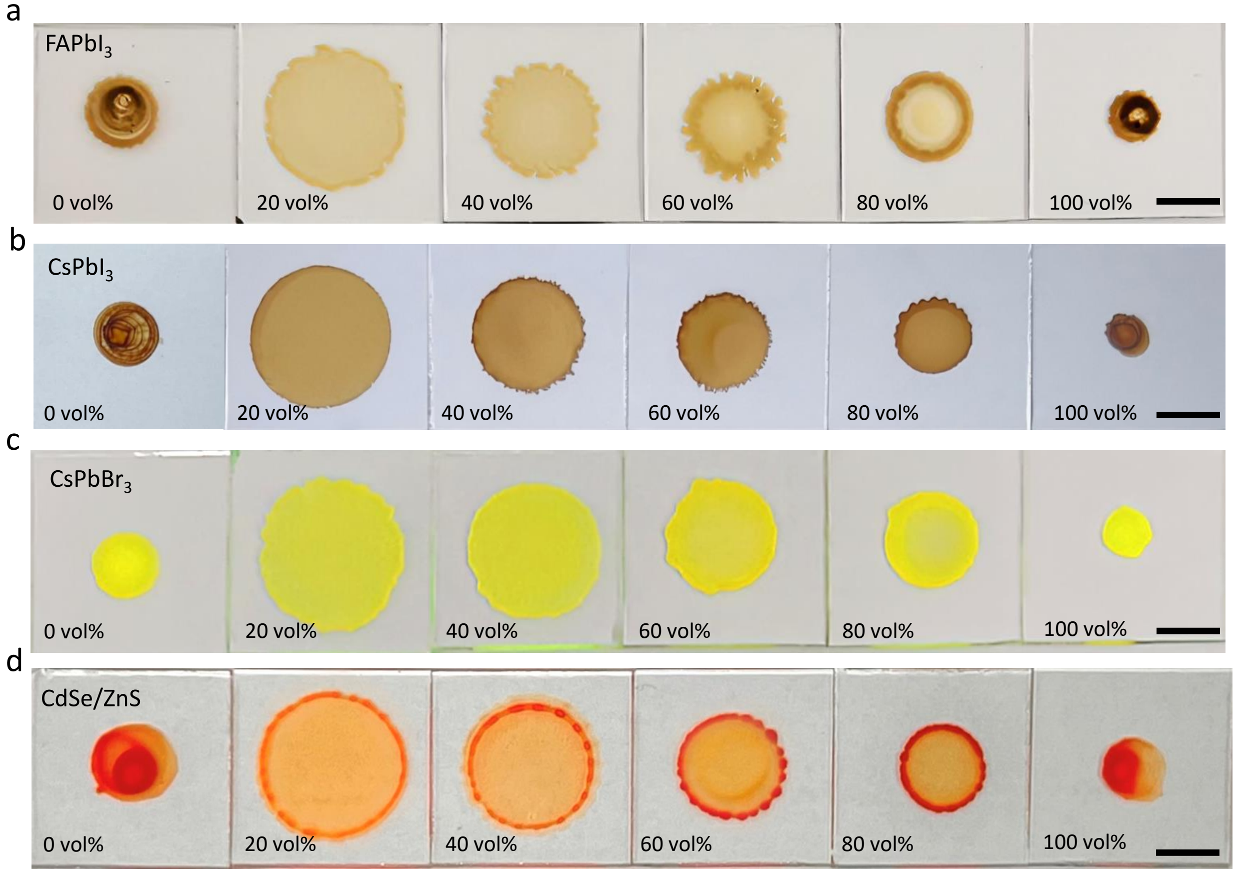

Supplementary Fig. 7 | Deposition behavior of quantum dot (QD) inks. a-d, Morphology of drop-cast $FAPbI_3$ (**a**), $CsPbI_3$ (**b**), $CsPbBr_3$ (**c**), and CdSe/ZnS (**d**) QD films with different n-hexane concentrations. Vol% is the volume percent. The volume of QD inks is 3 µl, and the scale bar is 1 cm.

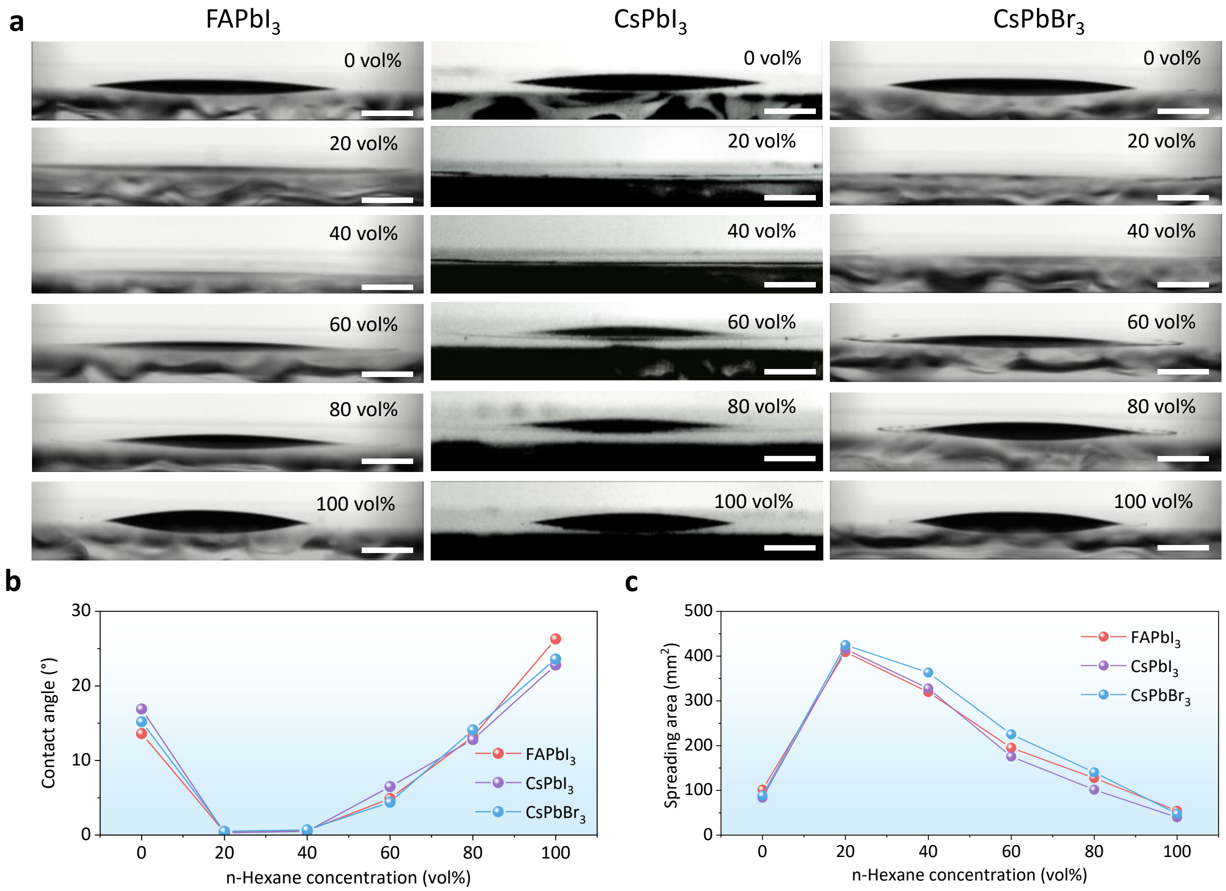

Supplementary Fig. 8 | Spreadability of perovskite quantum dot (PQD) inks. a, Contact angles of $FAPbI_3$, $CsPbI_3$, and $CsPbBr_3$ QD inks on glass substrates. The n-hexane volume ratio is marked in the figure. Vol% is the volume percent. **b,c,** Contact angle (**b**) and (**c**) spreading area of 3 µl inks of different PQDs with various n-hexane concentrations, and the scale bar is 2 mm.

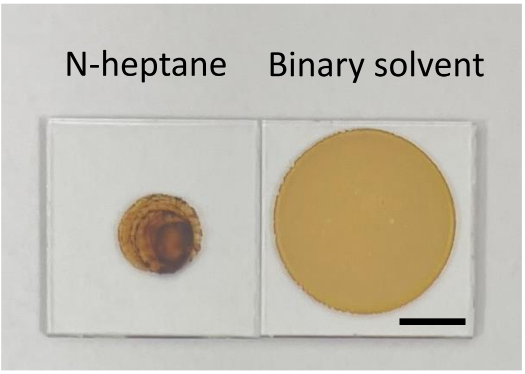

Supplementary Fig. 9 | Deposition behavior of perovskite quantum dot (PQD) inks with different solvents. Morphology of PQD films based on n-heptane and our binary solvent (20 vol% n-hexane). The volume of PQD inks is 3 μl, and the scale bar is 1 cm.

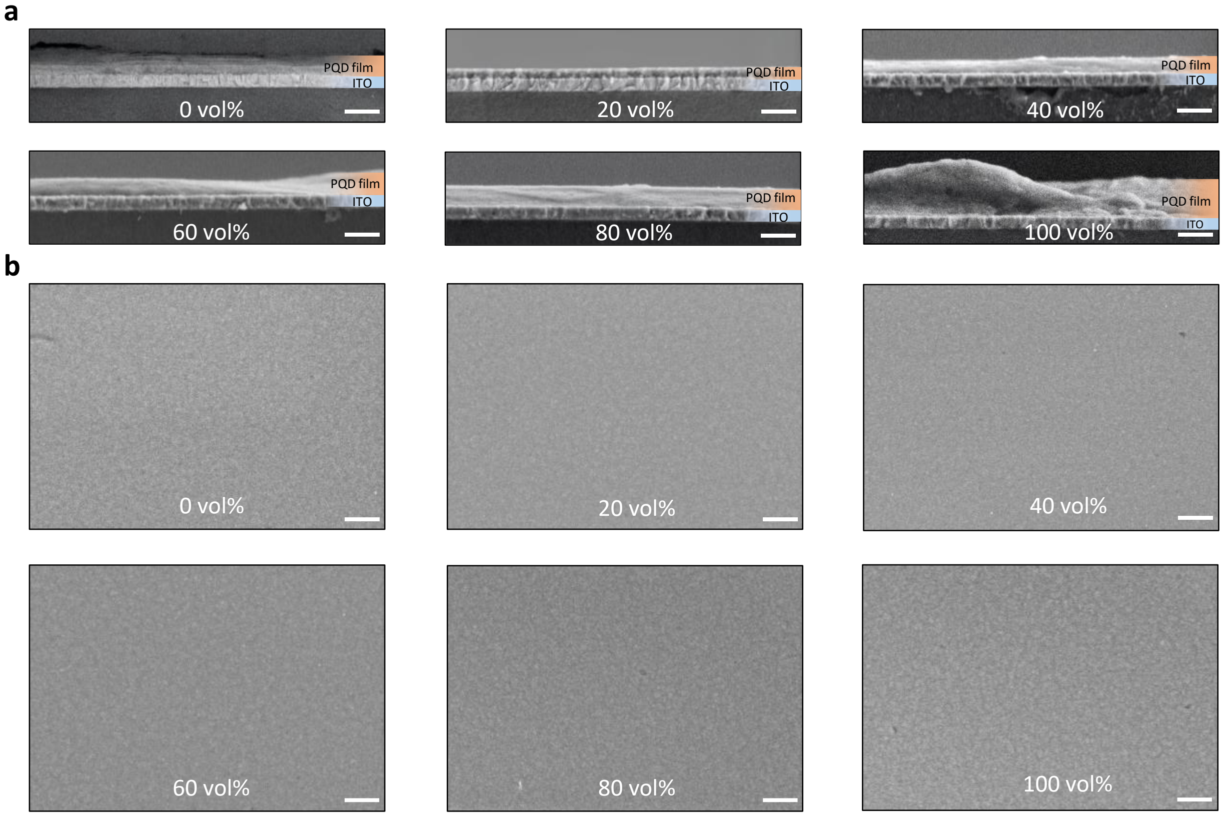

Supplementary Fig. 10 | Scanning electron microscopy (SEM) images of perovskite quantum dot (PQD) films. a,b, Cross-section (**a**) and surface SEM images (**b**) of drop-casted PQD films with different concentrations of n-hexane. Vol% is the volume percent. The scale bars are 200 nm.

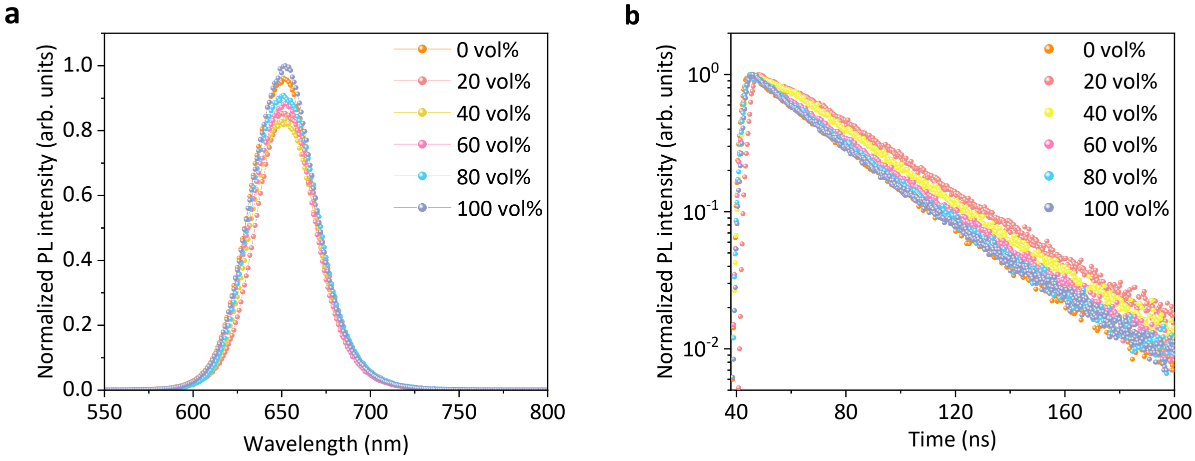

Supplementary Fig. 11 | Photoluminescence (PL) properties of perovskite quantum dot (PQD) films. a,b, Steady-state PL spectra (**a**) and transient PL decay curves (**b**) of PQD films with different n-hexane concentrations. The intensity of the steady-state PL spectra is normalized according to their photoluminescence quantum yield (PLQY) values. Vol% is the volume percent.

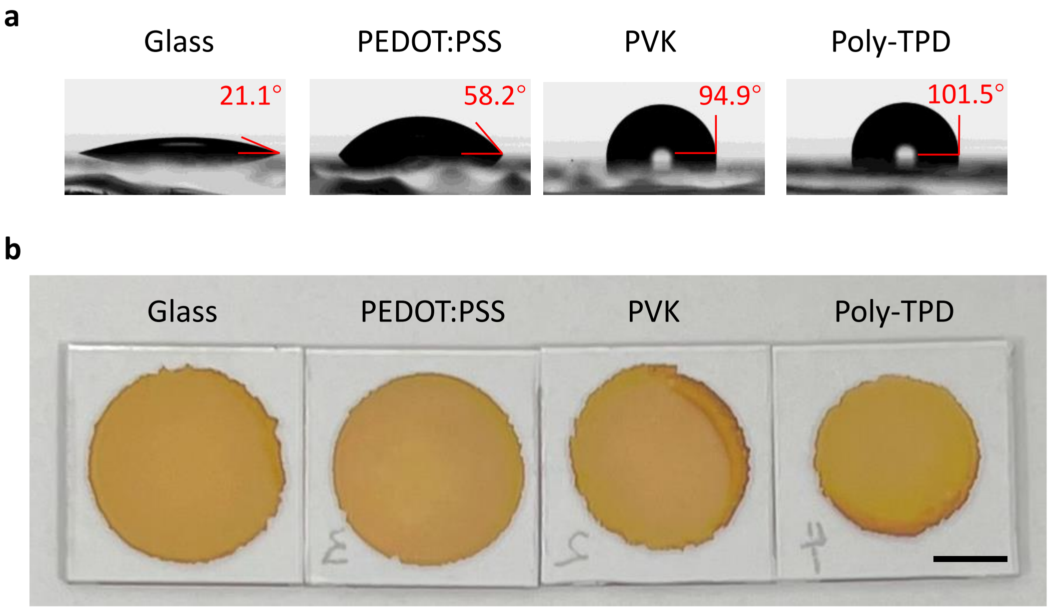

Supplementary Fig. 12 | Deposition behavior on different substrates. a,b, Contact angle of different substrates (**a**) and morphology (**b**) of perovskite quantum dot (PQD) films on substrates made using cosolvent with 20 volume percent (vol%) n-hexane. The volume of PQD inks is 3 μl, and the scale bar is 1 cm.

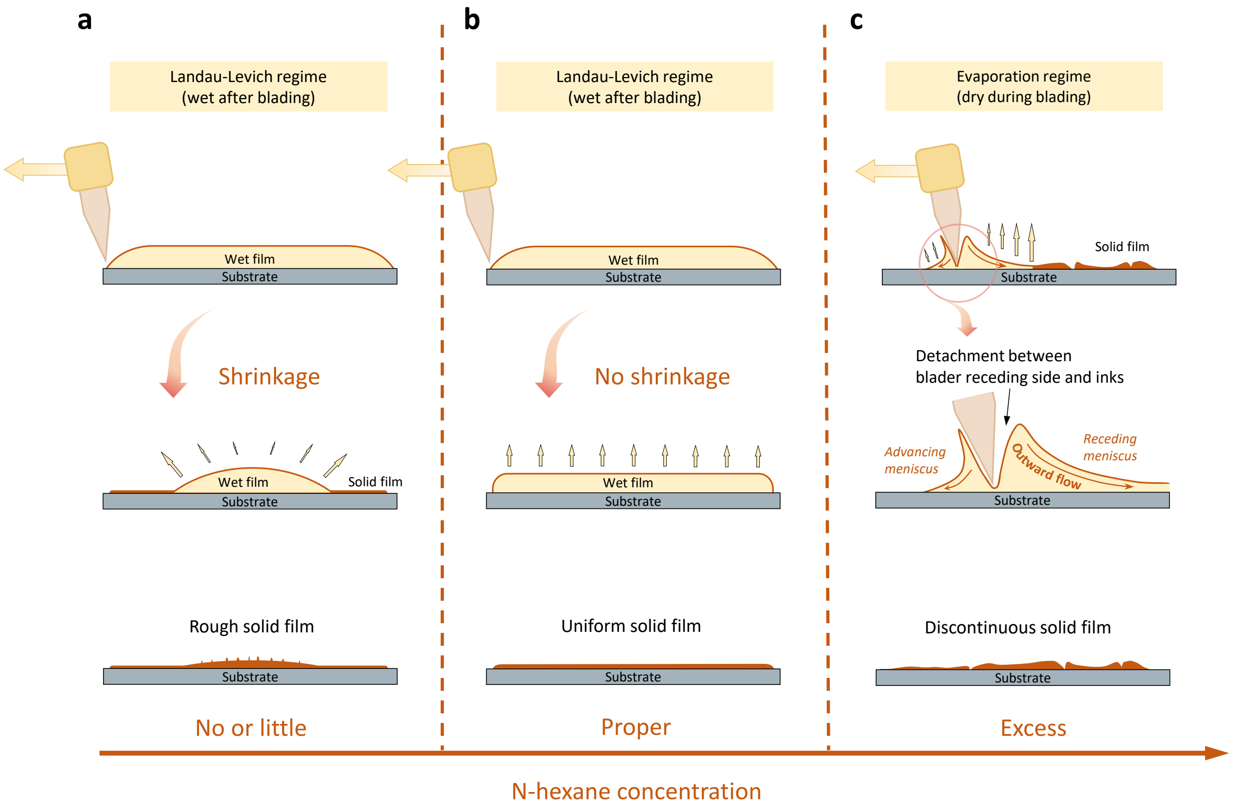

Supplementary Fig. 13 | Schematic illustration of the blade-coating regimes. a,b, Film-formation process in the Landau-Levich regime when the n-hexane concentration is no or little (**a**) and proper (**b**). **c,** Film-formation process in the evaporation regime when the n-hexane concentration is excess (**c**).

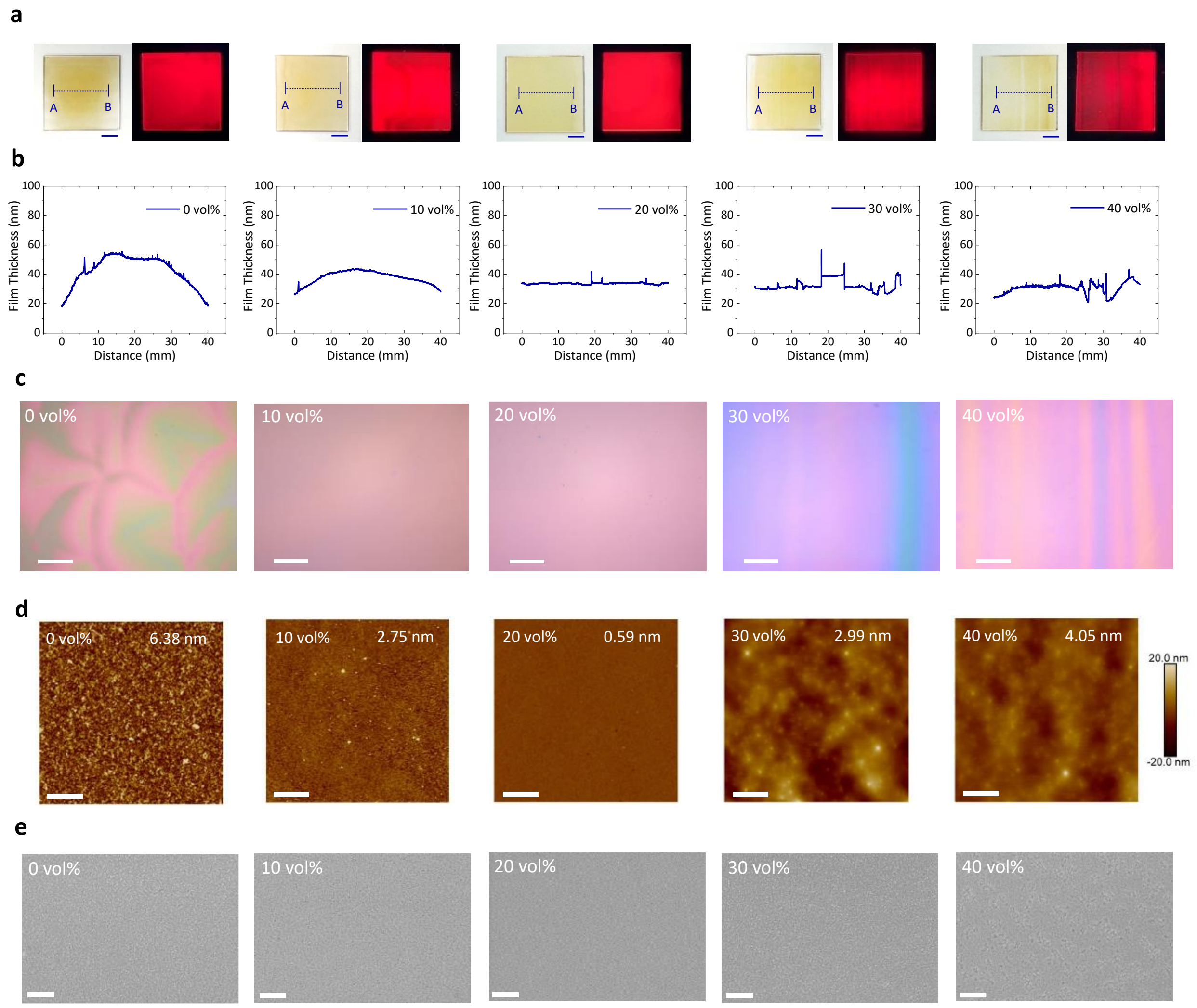

Supplementary Fig. 14 | Macroscale and microscale morphology characterizations of blade-coated perovskite quantum dot (PQD) films. a, Photograph of PQD films (5 cm × 5 cm) on a glass substrate under room light (left) and an ultraviolet (UV) lamp (right). The scale bar is 1 cm. **b,** Thickness profile of PQD films on the glass substrate as shown in (a) from "A" to "B". **c-e,** Optical microscopy (**c**), atomic force microscopy (AFM) (**d**), and scanning electron microscopy (SEM) (**e**) images of blade-coated PQD films with different n-hexane concentrations. Vol% is the volume percent. The scale bars are 100 µm, 5 µm, and 500 nm, respectively.

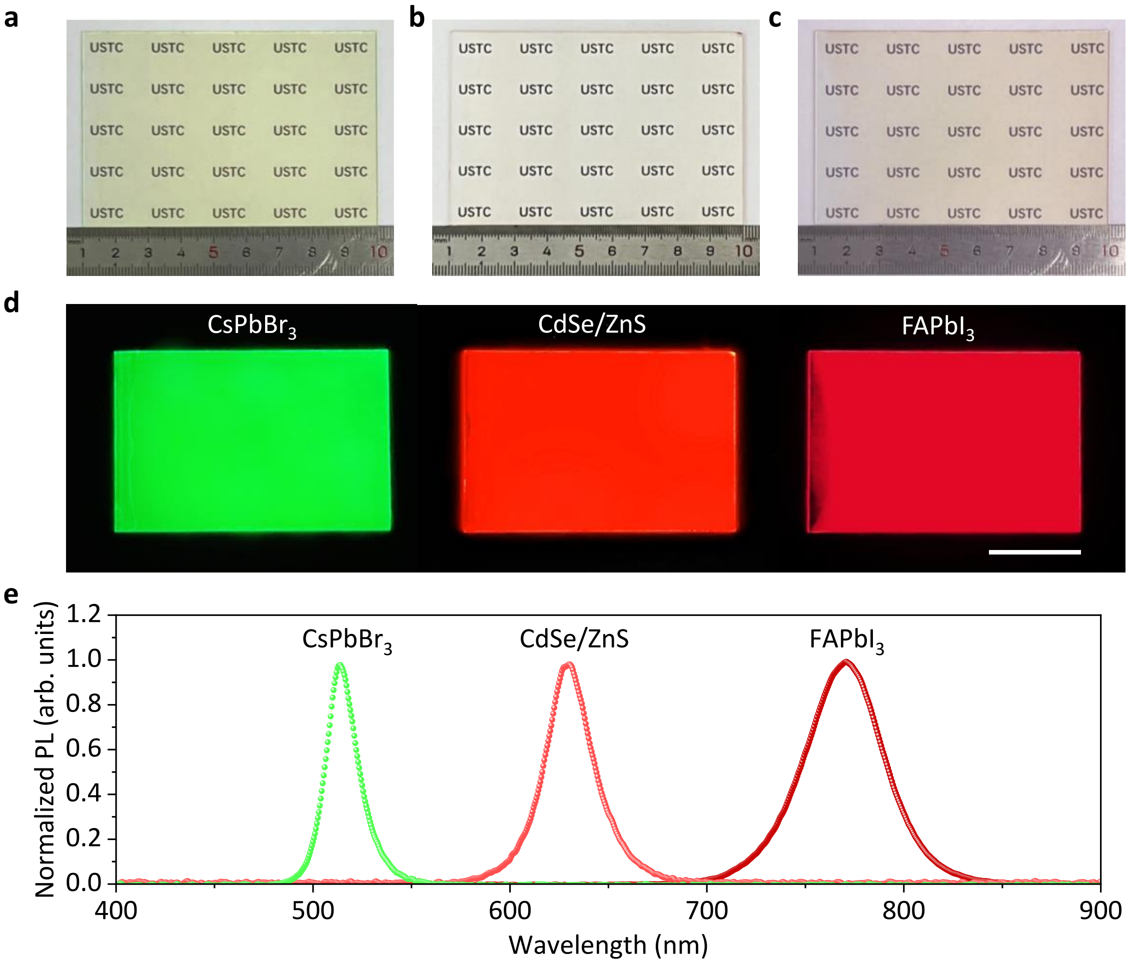

Supplementary Fig. 15 | Large-area quantum dot (QD) films with different compositions fabricated by blade coating. a-c, Photo image of 6 × 9 cm^2 blade-coated $CsPbBr_3$ (**a**), CdSe/ZnS (**b**), and $FAPbI_3$ (**c**) QD films. **d,e,** photoluminescence (PL) image (**d**) and PL spectra (**e**) of 6 × 9 cm^2 blade-coated $CsPbBr_3$, CdSe/ZnS, and $FAPbI_3$ QD films. The scale bar is 3 cm.

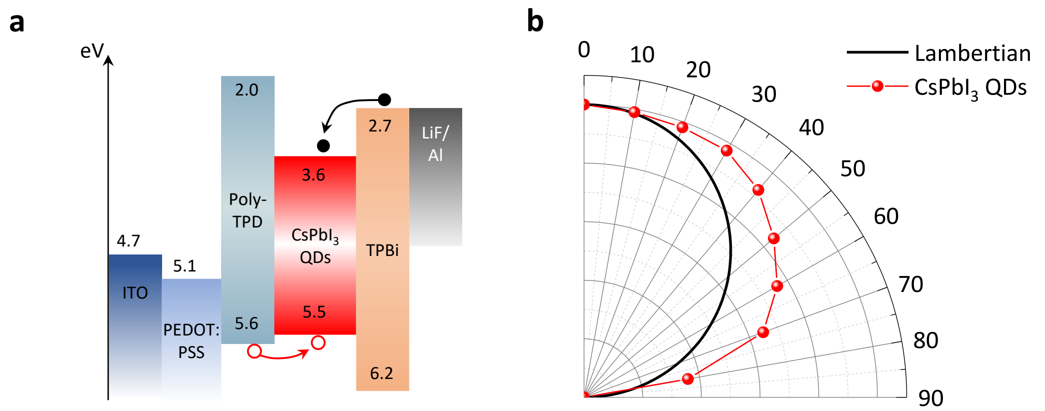

Supplementary Fig. 16 | Energy level and angular distribution. a,b, Energy diagram (**a**) and angular intensity profiles (**b**) of blade-coated perovskite light-emitting diodes (PeLEDs).

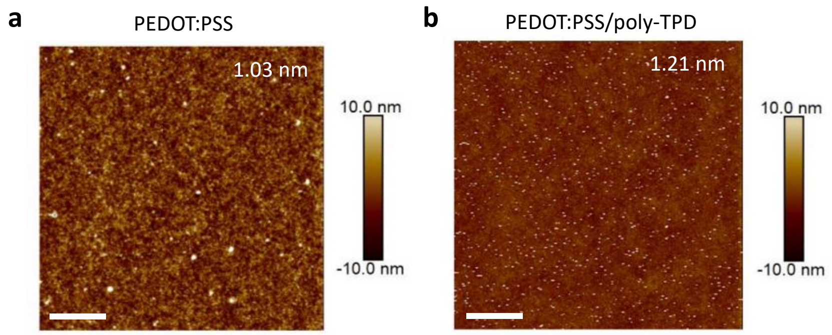

Supplementary Fig. 17 | Morphology of hole transport layers fabricated by blade coating. a, b, Atomic force microscopy (AFM) image of blade-coated poly(3,4-ethylenedioxythiophene): poly(styrenesulfonate) (PEDOT:PSS) (**a**) and PEDOT:PSS/poly(N,N'-bis(4-butylphenyl)-N,N'-bis(phenyl)-benzidine (poly-TPD) (**b**) layers. The surface roughness values are 1.03 nm and 1.21 nm, respectively. The scale bar is 5 μm.

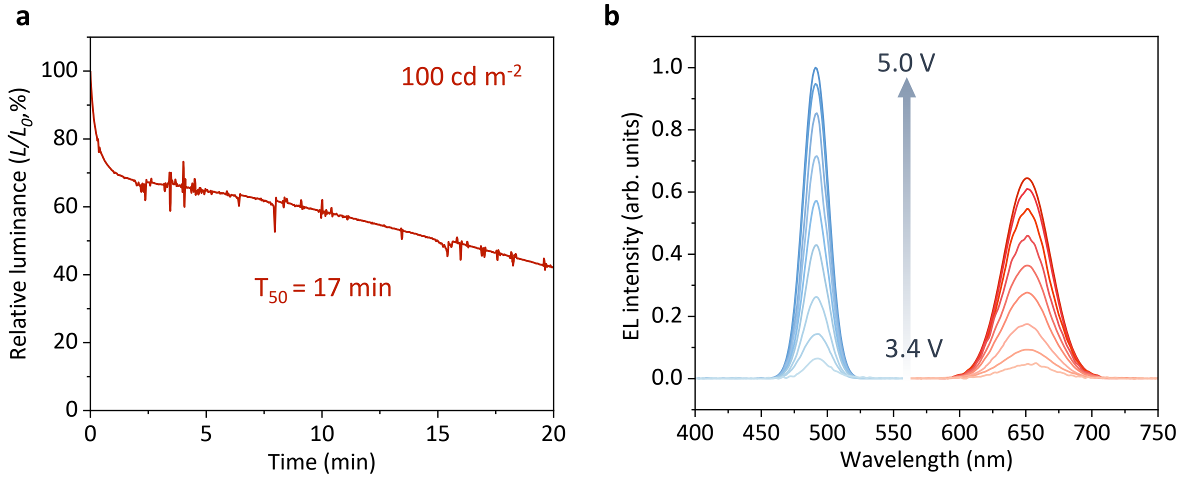

Supplementary Fig. 18 | Stability characterization of white perovskite light-emitting diodes (WPeLEDs). a, Device lifetime of WPeLEDs with a 4 mm^2 pixel area at an initial luminance of 100 cd m^{-2}. **b,** Spectral stability of WPeLEDs operating at different voltages.

Supplementary Table 1 | Optical properties of perovskite quantum dot (PQD) films. Summarized average carrier lifetime ($\tau_{average}$), photoluminescence quantum yield (PLQY), radiative recombination rate k_{rad} and nonradiative recombination rate k_{nonrad} of PQD films with different n-hexane concentrations.

	$\tau_{average}$ [ns]	PLQY [%]	k_{rad} [s^{-1}]	k_{nonrad} [s^{-1}]
0 vol%	34.4	34.6	1.0×10^7	1.9×10^7
20 vol%	43.5	46.8	1.1×10^7	1.2×10^7
40 vol%	41.8	44.6	1.1×10^7	1.3×10^7
60 vol%	40.7	42.4	1.0×10^7	1.4×10^7
80 vol%	38.5	41.9	1.1×10^7	1.5×10^7
100 vol%	34.9	35.1	1.0×10^7	1.9×10^7

Supplementary References

1 Ding, H., Spelt, P. D. M. & Shu, C. Diffuse interface model for incompressible two-phase flows with large density ratios. *J. Comput. Phys.* **226**, 2078-2095 (2007).

2 Sui, Y., Ding, H. & Spelt, P. D. M. Numerical Simulations of Flows with Moving Contact Lines. *Annu. Rev. Fluid Mech.* **46**, 97-119 (2014).
